# Serum Antimüllerian hormone does not predict elevated progesterone levels among women who undergo controlled ovarian hyperstimulation for in vitro fertilization

**DOI:** 10.1186/s12958-019-0477-8

**Published:** 2019-04-08

**Authors:** Shahryar K. Kavoussi, Shu-Hung Chen, Caitlin L. Hunn, Brady T. West, John David Wininger, Keikhosrow M. Kavoussi, Parviz K. Kavoussi

**Affiliations:** 1 Austin Fertility and Reproductive Medicine/Westlake IVF, 300 Beardsley Lane, Bldg B, Suite 200, Austin, TX 78746 USA; 2 Institute for Social Research, University of Michigan, Ann Arbor, MI 48109 USA

**Keywords:** AMH, Antimüllerian hormone, IVF, In vitro fertilization, Progesterone

## Abstract

Serum Antimüllerian hormone (AMH) has been shown to predict various in vitro fertilization (IVF) outcomes. AMH and progesterone (P) are products of granulosa cells of the ovary. Since overall granulosa cell number directly correlates with oocyte number and AMH production, the aim of this study is to evaluate whether or not serum AMH is associated with elevated P during controlled ovarian hyperstimulation (COH) for IVF. For this retrospective study, data were abstracted from charts of first IVF cycles of women (*n* = 201) who had undergone COH between May 2014 and May 2017. Groups were as follows: (A) AMH < 1 ng/mL (*n* = 32), (B) AMH 1–3.99 ng/mL (*n* = 109), (C), AMH ≥ 4 ng/mL (*n* = 60). The primary outcome measure was serum P level at trigger prior to oocyte retrieval. Mean serum P levels among groups A, B, and C were 0.92 ng/mL, 0.96 ng/mL, and 0.84 ng/mL, respectively. One-way ANOVA showed that there was no difference in mean serum P level among groups A, B, and C (*p*-value = 0.28). Multivariable linear regression with P as the dependent variable showed that total gonadotropin dose and peak estradiol level on day of trigger each had a significant positive relationship with P, and clinical pregnancy had a significant negative relationship. Although AMH is a predictor of certain IVF outcomes, AMH is not a predictor of elevated serum P level at trigger among women who undergo COH for IVF.

## Introduction

During the process of controlled ovarian hyperstimulation (COH) for in vitro fertilization (IVF) treatment, an elevated serum progesterone (P) level prior to oocyte retrieval may result from the multi-follicular development which is an inherent step prior to oocyte retrieval for IVF. This phenomenon may occur in up to 38% of COH cycles, despite the use of gonadotropin releasing hormone (GnRH) analogues [1–3]. Such an early rise in P level is associated with an advancement of the endometrial microarchitecture and the potential for embryonic-endometrial asynchrony [4, 5]. Although controversy exists as to whether or not an elevated serum P level on the day of exogenous follicle triggering has an effect on IVF outcomes [6], many studies, as well as systematic reviews and meta-analyses, have suggested that elevated P (≥ 1.5 ng/nl) is associated with lower pregnancy rates [7, 8] among women who undergo COH for IVF, and that a freeze-only of embryos is recommended in that cycle, in order to optimize outcomes by synchronizing the embryo and endometrium in a subsequent, more natural cycle [4].

Antimüllerian Hormone (AMH) is a member of the transforming growth factor (TGF)-β superfamily of glycoproteins and a widely-used serum biomarker to assess ovarian reserve in women undergoing COH for IVF. AMH is produced by ovarian granulosa cells which surround the primordial to pre-antral ovarian follicles in which oocytes are located [9]. AMH has been shown to be predictive of oocyte yield at retrieval as well as other IVF outcomes [10–13], and this biomarker has been used to optimize COH protocols that employ exogenous gonadotropins for IVF treatment cycles [14].

Since both the steroid hormone P and the glycoprotein AMH are products of granulosa cells [15] and since AMH has been found to be a predictor of several IVF outcomes, the aim of this study is to determine whether AMH predicts the premature P rise prior to oocyte retrieval. If an association exists, a potential mechanism of action may be elucidated to explain elevated P and, in cases of intended fresh embryo transfer, patients may be counseled about the likelihood a freeze-only of embryos due to elevated P levels, prior to starting COH for IVF.

## Methods

A retrospective chart review was performed, with data abstracted from first IVF cycles of women (*n* = 201) who had undergone COH at Austin Fertility & Reproductive Medicine/Westlake IVF between May 2014 and May 2017. Inclusion criteria were females between the ages of 18 and 44 years, all causes of infertility, and cases of COH with exogenous gonadotropins. The exclusion criteria were initial intent of IVF for freeze-only either due to preimplantation genetic testing (PGT) or from an elective standpoint, COH with oral ovulation induction agents or a combination of oral ovulation induction agents with exogenous gonadotropins, as well as donor oocyte/IVF and donor embryo cases. Due to the de-identified nature of the data collected, Institutional Review Board exemption was obtained from St. David’s Healthcare Institutional Review Board.

An AMH level > 1.0 ng/mL, regardless of day of the cycle, was considered as a normal level. Group (A) consisted of women with AMH < 1 ng/mL (*n* = 32), Group (B) was that of women with AMH of 1–3.99 ng/mL (*n* = 109), and Group (C) was comprised of women with AMH ≥ 4 ng/mL (*n* = 60). A high AMH threshold of 4 mg/mL was used in this study because, in our patient population, a patient is more likely to have a significantly greater ovarian response. In addition, the authors of the following article indicate that an increased risk of severe ovarian hyperstimulation syndrome, consistent with excessive ovarian response, is generally seen in women with AMH level of 4–5 ng/mL [16]. The AMH level was measured (Ansh laboratories, Webster, TX) for each patient within proximity to (at most within 6 months) COH for IVF. Individualized stimulation protocols with gonadotropin dosage and gonadotropin releasing hormone (GnRH) analogues based on patient AMH level and age were used, including the GnRH antagonist protocol which all subjects in Groups B and C used as well as the majority of subjects in Group A used as two subjects in that groups used the GnRH-agonist microdose flare protocol. During ovarian stimulation with exogenous gonadotropins, serial transvaginal sonograms as well as serum estradiol levels were monitored, with P levels (Roche COBAS® Electrochemiluminescent immunoassay run by Clinical Pathology Laboratories, Austin, TX) being monitored as well, after lead follicle(s) measured 16 mm or greater in average diameter. After ovarian stimulation and subsequent transvaginal ultrasound-guided oocyte retrieval 35–36 h after human Chorionic Gonadotropin (hCG) administration, either IVF or intracytoplasmic sperm injection (ICSI) was performed. Embryo transfer was performed on day 3 or day 5 based on embryo quality and in accordance with the American Society for Reproductive Medicine (ASRM) /Society for Assisted Reproductive Technology (SART) guidelines for the number of embryos to be [17]. Surplus good quality blastocysts were cryopreserved on day 5 or day 6.

The primary outcome measure was serum P level on the day of trigger prior to oocyte retrieval. Secondary outcome measures were number of days of COH, total gonadotropin dosage during COH, number of follicles > 14 mm in average diameter on the day of trigger, estradiol level on the day of trigger, number of M2 oocytes at retrieval, and number of 2 pronuclear (2PN) zygotes. Data were expressed as means ± SD. Statistical analyses were performed with one-way ANOVA and multivariable linear regression. *P*-value < 0.05 was considered as statistically significant.

## Results

The incidence of elevated progesterone level on the day of trigger for Groups A, B, and C were 15.6% (5/32), 12.8% (14/109) and 1.7% (1/60), respectively (Chi square: Groups A vs B, *p* = 091; Group A vs C, *p* = 0.03; Groups B vs C, p = 0.03). Characteristics and IVF outcomes of women in Group A, B, and C are shown in Table 1. In terms of the primary outcome measure, mean serum P levels among groups A, B, and C were 0.92 ng/mL, 0.96 ng/mL, and 0.84 ng/mL, respectively; there was no statistical difference in mean serum P level among groups A, B, and C (*p*-value = 0.28). In terms of secondary outcome measures, although there were no differences among groups in for number of days of COH (*p*-value = 0.73), there were statistically significant differences among groups for total gonadotropin dosage during COH (*p*-value < 0.01), number of follicles > 14 mm in average diameter on the day of trigger (*p*-value < 0.01), estradiol level on the day of trigger (p-value < 0.01), number of M2 oocytes at retrieval (p-value < 0.01), and number of 2PN zygotes (p-value < 0.01).

**Table 1:** Variables including demographics and outcomes for study group Group A (women with AMH <1 ng/mL), study Group B (women with AMH 1–3.99 ng/mL), and study Group C (women with AMH ≥ 4 ng/mL)

Characteristic	Group A (*n* = 32) AMH < 1 ng/mL	Group B (*n* = 109) AMH 1–3.99 ng/mL	Group C (*n* = 60) AMH ≥ 4 ng/mL	*P*-value
Age (years)	36.9 +/− 3.81	34.2 +/− 4.00	31.3 +/− 3.85	< 0.01
AMH (ng/mL)	0.576 +/− 0.28	2.258 +/− 0.89	6.44 +/− 2.44	< 0.01
Basal E2 (pg/mL)	21.47 +/− 12.07	19.50 +/− 8.59	23.72 +/− 11.01	0.03
Basal AFC	11.32 +/− 3.64	17.43 +/− 6.37	25.70 +/− 7.46	< 0.01
No. of days of COH	9.59 +/− 1.48	9.62 +/− 1.48	9.433 +/− 1.56	0.73
Total Gn dose (IU)	3464.06 +/− 845.41	2611.24 +/− 633.16	1838.33 +/− 636.15	< 0.01
# follicles ≥ 14 mm^a^	5.06 +/− 2.28	9.96 +/− 4.26	15.233 +/− 6.29	< 0.01
E2 (pg/mL) at trigger	1090.20 +/− 485.52	1876.66 +/− 696.30	2371.84 +/− 890.07	< 0.01
P4 (ng/mL) at trigger	0.92 +/− 0.60	0.96 +/− 0.46	0.84 +/− 0.39	0.28
# M2 oocytes retrieved^b^	4.33 +/− 3.47	9.06 +/− 4.51	12.83 +/− 5.81	< 0.01
# of 2PN zygotes^c^	3.59 +/− 2.45	6.43 +/− 3.96	8.90 +/− 5.26	< 0.01
CPR (per fresh ET)	33.3% (8/24)	52.2% (48/92)	74.5% (38/51)	< 0.01

Data are expressed as means +/− SD, and were analyzed via ANOVA*, with AMH as the independent variable for dependent variables/outcomes with the exception of CPR which is expressed as percentages and was analyzed via Fisher’s Exact test. *P*-value < 0.05 was considered statistically significant

*AMH* = Antimüllerian hormone

*AFC* = antral follicle count

*COH* = controlled ovarian hyperstimulation

*CPR* = clinical pregnancy rate

*E2* = estradiol

*ET* = embryo transfer

*Gn* = gonadotropin

*M2* = metaphase II

*P4* = progesterone

*PN* = pronuclear

^a^Group A (*n* = 31)

^b^Group A (*n* = 30), Group B (*n* = 108); Group C (*n* = 58)

^c^Group A (*n* = 27), Group B (*n* = 107); Group C (n = 58)

A multivariable linear regression model, with P as the dependent variable and independent variables shown in Table 2, was fitted, with 166 cases analyzed due to missing data on the covariates. Using a *p* <  0.05 significance level, total gonadotropin dose and peak estradiol level at trigger had significant positive relationships with P, and clinical pregnancy had a significant negative relationship with P, adjusting for all of the other covariates (Table 2). Based on the fitted model, group B had a significantly higher mean than group A (*p* = 0.024) when adjusting for the other covariates, but overall, group is only marginally significant as a factor (*p* = 0.07, based on a Wald test for the two group parameters).

**Table 2:** Multivariate linear regression model using progesterone (P4) as the dependent variable for the listed independent variables

Independent variable	Progesterone (P4)		
	Coefficient (standard errors)	*P* value	95% C.I.
Age (years)	−0.00720 (0.00718)	0.316	(−0.214, 0.007)
AMH (ng/mL)	−0.01418 (0.02127)	0.506	(−0.056, 0.028)
Basal E2 (pg/mL)	0.00106 (0.00276)	0.701	(−0.044, 0.007)
Basal AFC	−0.00353 (0.00433)	0.416	(−0.012, 0.005)
No. of days of COH	−0.02756 (0.02461)	0.262	(−0.076, 0.208)
Total Gn dose (IU)	0.00017 (0.00006)	0.004 *	(0.000, 0.003)
# follicles ≥ 14 mm^a^	−0.00301 (0.00888)	0.735	(−0.205, 0.015)
E2 (pg/mL) at trigger	0.00001 (0.00005)	0.044 *	(0.000, 0.000)
# M2 oocytes retrieved^b^	0.01693 (0.01208)	0.163	(−0.007, 0.041)
# of 2PN zygotes^c^	0.00689 (0.01218)	0.573	(−0.172, 0.031)
CPR (per fresh ET)	−0.10756 (0.05308)	0.044 *	(−0.212, − 0.003)

*AMH* = Antimüllerian hormone

*AFC* = antral follicle count

*COH* = controlled ovarian hyperstimulation

*CPR* = clinical pregnancy rate

*E2* = estradiol

*ET* = embryo transfer

*Gn* = gonadotropin

*M2* = metaphase II

*P4* = progesterone

*PN* = pronuclear

## Discussion

AMH has been shown to be a strong predictor of ovarian response to exogenous gonadotropins during COH for IVF as well as outcomes such as oocyte yield [18], blastocyst formation [19], blastocyst aneuploidy [20], and surplus embryo cryopreservation at the various stages [21–26]. There are conflicting data regarding the ability of AMH to predict clinical pregnancy rates and live birth rates [27–31].

Freeze-all cycles are more common in recent years and have been instrumental in improving overall IVF and obstetric outcomes; however, there are data that support fresh embryo transfer in subsets of patients [32, 33]. In such cases of patients who initially opt for fresh embryo transfer, the inquiry as to the likelihood of freeze-all due to an elevated progesterone level is sometimes made. A previous publication had found that serum P level on day of trigger was associated with serum estradiol on the day of trigger, the number of follicles > 14 mm, and the number of oocytes retrieved. The authors found that basal levels of neither follicle stimulating hormone nor estradiol were associated with elevated P; however, AMH was not studied as a potential predictor [34]. Since the glycoprotein AMH is produced by granulosa cells of the ovary and since the steroid hormone P is also a product of granulosa cells, we hypothesized that AMH may predict elevated P levels during the late follicular phase of COH with pituitary suppression of LH.

In our study, a one-way ANOVA showed no difference in mean serum P levels among women grouped into those with AMH < 1 ng/mL, AMH 1–3.99 ng/mL and AMH ≥ 4 ng/mL. Subsequent multivariable linear regression showed that total gonadotropin dose and peak estradiol level at trigger each had a positive relationship with P level, and clinical pregnancy had a negative relationship with P level. There was weak evidence of group differences based on this model when adjusting for the other covariates, and these differences may become more apparent in future studies with larger sample sizes.

Limitations of our study include the retrospective nature with the inherent biases with such study design. Another study limitation was the sample size, particularly in the low AMH group (Group A). Basal AMH levels were not checked amongst the patients, which may be considered a minor limitation of this study and may be a potential area to explore in future studies. Although there may have been concerns about luteal deficiency in PCOS, this has predominantly been in the scenario of ovulation induction instead of the COH protocols used for IVF. A previous study, which involved IVF patients, showed a higher mean progesterone level on the day of trigger among women with PCOS when compared to women without PCOS [35]. Strengths of our study include the use of GnRH antagonist protocols for the vast majority of cases (94% of subjects in Group A, 100% of subjects in Group B, and 100% subjects in Group C) as well as the use of one laboratory for measurement of P level. In addition, analysis of this study’s data set seemed consistent with the existing literature in terms of showing that AMH was associated with established factors such as ovarian response to exogenous gonadotropins and oocyte yield at retrieval, with there being no difference amongst groups in terms of progesterone level on the day of trigger.

## Conclusion

We conclude that although AMH is a predictor of certain IVF outcomes, AMH is not a predictor of elevated serum P level at trigger among women who undergo COH for IVF. Although larger studies are necessary due to the limitations of the retrospective study design and sample size of subjects, these data suggest that the mechanism of action of elevated P in COH cycles is unlikely to be due to directly proportionate granulosa cell numbers, and from a clinical standpoint, AMH cannot be used to counsel patients of the likelihood of a freeze-all cycle due to elevated P if their initial intent is fresh embryo transfer.

## Abbreviations

AMH: antimüllerian hormone
ASRM: American Society for Reproductive Medicine
COH: controlled ovarian hyperstimulation
GnRH: gonadotropin releasing hormone
hCG: human Chorionic Gonadotropin
ICSI: intracytoplasmic sperm injection
IVF: in vitro fertilization
P: progesterone
PGT: preimplantation genetic testing
PN: pronuclear
SART: Society for Assisted Reproductive Technology
TGF-β: transforming growth factor-β
